# Neonatal prophylaxis with antibiotic containing ointments does not reduce incidence of chlamydial conjunctivitis in newborns

**DOI:** 10.1186/s12879-021-05974-3

**Published:** 2021-03-17

**Authors:** Tamar A. Smith-Norowitz, Crystal Ukaegbu, Stephan Kohlhoff, Margaret R. Hammerschlag

**Affiliations:** 1 Department of Pediatrics, State University of New York Downstate Medical Center, 450 Clarkson Ave., Box 49, Brooklyn, NY 11203 USA; 2 School of Public Health, State University of New York Downstate Medical Center, New York, Brooklyn USA

**Keywords:** Neonatal chlamydial prophylaxis, *Chlamydia trachomatis*, Silver nitrate, Erythromycin, Tetracycline

## Abstract

**Background:**

Neonatal ocular prophylaxis with silver nitrate does not prevent neonatal conjunctivitis due to *Chlamydia trachomatis*. The efficacy of antibiotic containing preparations for prevention of neonatal chlamydial conjunctivitis (NCC) has not been established.

**Objective:**

To examine published literature to determine whether antibiotic containing preparation are efficacious for prevention of NCC and *C. trachomatis* in the nasopharynx.

**Methods:**

A literature search of MEDLINE and EMBASE. Articles were selected for review if their content included 4 key criteria: (1) Prospective/comparative study. (2) Prenatal screening of mothers for *C. trachomatis* with results reported. (3) Follow-up of infants born to chlamydia-positive women. (4) Infants prospectively followed at regular intervals and tested for *C. trachomatis* in the eye/ nasopharynx (NP).

**Results:**

The search yielded 159 studies; 11 were selected for full reviews, eight were excluded; three addressed the four criteria. Rates of *C. trachomatis* conjunctivitis in infants in included studies who received silver nitrate was 20–33%; positive NP, 1–28% and pneumonia, 3–8%. Rates of *C. trachomatis* conjunctivitis in neonates who received erythromycin or tetracycline prophylaxis did not differ from silver nitrate; 0–15 and 11%, respectively, who received erythromycin or tetracycline developed NCC. Similarly, 4–33 and 5% of infants who received erythromycin or tetracycline, respectively, had positive NP cultures; 0–4% developed chlamydial pneumonia.

**Conclusion:**

Neonatal ocular prophylaxis with erythromycin or tetracycline ophthalmic ointments does not reduce incidence of neonatal chlamydial conjunctivitis or respiratory infection in infants born to mothers with *C. trachomatis* infection compared to silver nitrate.

## Introduction

Credé reported in 1881 that instillation of 2% silver nitrate drops into the eyes of newborn infants reduced the incidence of gonococcal ophthalmia neonatorum [1]. However, the epidemiology of ophthalmia neonatorum has significantly changed since 1881. Gonococcal ophthalmia is very uncommon mainly due to prenatal screening for *Neisseria gonorrhoeae* and treatment of pregnant women [2, 3]. *C. trachomatis* was the most common cause of neonatal conjunctivitis in the U.S. before the Centers for Disease Control and Prevention (CDC) recommended routine prenatal screening of pregnant women for *C. trachomatis* in 1993 [2]. Currently, erythromycin ophthalmic ointment is the only preparation available for neonatal ocular prophylaxis in the U.S. [4]; tetracycline ophthalmic ointment is no longer manufactured, and silver nitrate has not been available in the U.S. for over two decades.

Prenatal screening and treatment of pregnant women for chlamydia has resulted in a decrease in neonatal chlamydial conjunctivitis and pneumonia [4–6]. Prospective studies of vertical transmission of *C. trachomatis* conducted from the 1970s to 1980s found that neonatal ocular prophylaxis with silver nitrate did not appear to prevent chlamydial ophthalmia or nasopharyngeal (NP) colonization with *C. trachomatis* or chlamydial pneumonia [7–11]. A study published by Hammerschlag, et al in 1980 suggested that neonatal ocular prophylaxis with erythromycin ointment was effective in prevention of chlamydial conjunctivitis but did not reduce NP infection or pneumonia [12]. However, a subsequent study [3] demonstrated that neonatal ocular prophylaxis with either erythromycin or tetracycline ophthalmic ointment did not significantly reduce the incidence of chlamydial conjunctivitis in infants of mothers with chlamydial infection as compared with silver nitrate [3]. Many hospitals in the US switched to erythromycin ophthalmic ointment after the initial study by Hammerschlag et al [12]. The law in many states also specifically mandates neonatal ocular prophylaxis with erythromycin.

The CDC currently recommends neonatal ocular prophylaxis with erythromycin ophthalmic ointment, primarily for prevention of gonococcal ophthalmia [13]. The World Health Organization (WHO) still recommends neonatal ocular prophylaxis for prevention of both gonococcal and chlamydial ophthalmia [14]. The aim of the current study was to review the published literature on whether antibiotic containing preparations are effective in prevention of neonatal chlamydial conjunctivitis.

## Methods

### Search strategy and selection criteria: literature search methods

A review was conducted according to Preferred Reporting Items for Systematic Reviews and Meta-Analysis (PRISMA) protocol guidelines. The PRISMA Protocols consists of a 17-item checklist that facilitates preparing and reporting a protocol for the systematic review.

### Eligibility criteria. (inclusion criteria)

Studies were eligible for inclusion if they included our four key criteria: (1) prospective study and comparative study, (2) prenatal screening of the mothers for *C. trachomatis* with results reported, (3) follow-up of infants born to *C. trachomatis*-positive women, and (4) infants were seen at regular intervals, cultured/tested for *C. trachomatis* in the eye/ nasopharynx (NP) whether or not they were symptomatic, and ointments containing antibiotic applied preventatively.

#### Information sources and search strategy

Electronic databases, including MEDLINE and EMBASE were used to identify relevant English language articles. Published literature in a wide range of styles and formats were selected for review, after working with a research librarian to develop our search strategy. There were no restrictions on country of origin, type of hospital, study type, journal type or year published. Articles were selected at each level of review due to their inclusion of content that matched with our four key criteria.

The Initial EMBASE search terms included:

1. chlamydia AND prophylaxis AND infant
2. “Ophthalmic Solutions”[Mesh] OR (“Chlamydia”[Mesh] OR “Chlamydia Infections”[Mesh]) AND (Randomized Controlled Trial [ptyp] AND “infant”[MeSH Terms])
  The last search date was performed on July 30, 2019.

Study records.

**Fig. 1:**
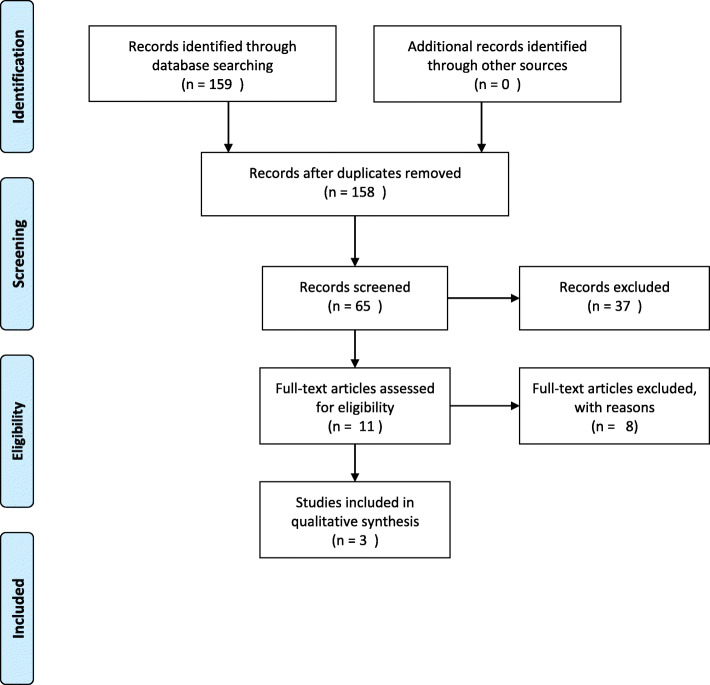
Initial study selection. Prisma Flow Diagram details our search and selection process applied during the overview

- Data management. Prisma Admin (data management tool)
- Selection process. Three independent researchers selected articles from the initial list of titles and abstracts to conduct a full review (Fig. 1). The full text articles were independently assessed for inclusion eligibility (i.e. eligible for further review).
- Data Collection process. Results of studies were not combined. Data collection was done independently.

##### Risk of bias in individual studies

The risk of bias was assessed in all studies by three independent review authors. Each reviewer then recorded his or her findings on a separate ‘Bias Assessment Form’. The form includes risk of bias, confounding and precision. Overlapping concerns of bias were then selected for final review and incorporation into the literature review. The concerns wholly addressed bias at the study level. All data were portrayed independently.

### Data synthesis and abstract review

Details of the studies that met the inclusions criteria listed above were then reviewed, summarized, and categorized according to the four key criteria. Three authors independently reviewed each group of abstracts identified by the literature search. A full text article was retrieved in cases in which the reviewers could not determine whether the article met the eligibility criteria from the abstract alone.

### Confidence in cumulative evidence

#### Statistical methods

Exact logistic regression was used to estimate exact odds ratios (ORs) with 95% confidence intervals (CIs); and to compute exact 2-sided tests of the null hypothesis that OR = 1. All statistical analyses were performed at the Scientific Computing Center (SUNY Downstate Medical Center, Brooklyn, NY).

#### Availability of data and materials

The data sets supporting the conclusions of this article are included within this article.

## Results

### Description and methodology of studies

The initial search yielded 159 unique published studies; 11 were selected for full-text review, eight were excluded because they did not meet all four key criteria (Fig. 1). Three studies were eligible for inclusion (Fig. 1). Excluded studies are summarized in Table 1, and included studies are summarized in Table 2.

**Table 1:** Characteristics of excluded studies

Study, year, location, ref. #	Prospective	Was study comparative?	Ocular prophylaxis Preparations	Prenatal screening for *C. trachomatis*	Number of Infants	Infants followed prospectively	Only Infants with conjunctivitis tested for *C. trachomatis*	All infants seen prospectively, and tested for *C. trachomatis* in eye and NP
1. Laga (1988) [15], Kenya	Yes	Yes	Silver nitrate Tetracycline	No*	1233 1499	Yes	Yes	Yes
2. Chen (1992) [16], Taiwan	Yes	Yes	Silver nitrate Tetracycline Erythromycin No prophylaxis Erythromycin twice	No	1082 1156 1163 1143 425	No	No	No
3. Rours (2006) [17], South Africa	Yes	No#	Tetracycline	Yes	77	Yes	Yes	Yes
4. Ali (2007) [18], Iran	Yes	Yes	Betadine Erythromycin No prophylaxis	No	110 110 110	Yes	Yes	No
5. Matinzadeh (2007) [19], Iran	Yes	Yes	Erythromycin No prophylaxis Normal saline	No	320 337 335	Yes	Yes	Yes
6. Ramirez-Ortiz (2007) [20], Mexico	Yes	Yes	Povidone-iodine vs. chloramphenicol	No	2004	Yes	Yes	Yes
7. David (2011) [21], Israel	Yes	No	Povidone iodine vs. tetracycline	No	394	Yes	Yes	No
8. Alexandre (2015) [22], Angola	No	No	Povidone-iodine vs. no prophylaxis	No	317	No	No	No

* Women identified intrapartum, results not available at delivery. # No comparative arm

1% silver nitrate drops

1% tetracycline ophthalmic ointment

0.5% erythromycin ophthalmic ointment

2.5% povidone-iodine drops

**Table 2:** Characteristics of included studies

	Hammerschlag (1980) [12]	Bell (1987) [23]	Hammerschlag (1989) [3]
**Location**	Seattle, WA	Seattle, WA	Brooklyn, NY
**Type of Study**	Prospective, randomized	Prospective, not randomized	Prospective, randomized
**Prophylaxis**	a. Silver Nitrate, b. Erythromycin	a. Silver Nitrate, b. Erythromycin	a. Silver Nitrate, b. Erythromycin, c Tetracycline
**Number of Infants**	60	120	230
**Chlamydial Conjunctivitis Number (%)**	a. 12 (33) b. 0	a. 21 (23) b. 4 (15)	a. 15 (20) b. 13 (14) c. 7 (11)
**NP Number (%)**	a.10 (28) b. 5	NR#	a. 1 (1) b. 4 (4) c. 3 (5)
**Pneumonia Number (%)**	a. 3 (8) b. 1 (4)	NR	a. 2 (3) b. 0 c. 0

a Silver nitrate; b. Erythromycin; c. Tetracycline

# Extraocular chlamydial infection without conjunctivitis. Sites tested NP, oropharynx, vagina and anus.

*NR* Not reported.

### Excluded studies

Of the eight excluded studies, one was not prospective [22] and seven did not perform prenatal screening for *C. trachomatis* [15, 16, 18–22]. Two of the studies did not follow all the infants prospectively; only infants who returned with conjunctivitis were tested for *C. trachomatis* [16, 22]. Infants were followed prospectively in four of the studies but were not tested for *C. trachomatis* in the eye and NP [16, 18, 21, 22].

Ali, et al*,* in a study from Iran [18], compared Betadine, erythromycin and no prophylaxis in 330 infants [18]; however, mothers were not tested for *N. gonorrhoeae* or *C. trachomatis* before delivery [18]. Matinzadeh, et al, also from Iran [19], compared erythromycin to saline in 1002 infants [19]; mothers were also not screened for *N. gonorrhoeae* or *C. trachomatis* [19]. Ramirez-Ortiz, et al*,* in a study from Mexico [20], compared 2.5% povidone-iodine and topical chloramphenicol in 2004 infants [20]; mothers were not screened prenatally. David, et al in a study from Israel of 394 full-term neonates compared povidone-iodine to tetracycline ophthalmic ointment [21]; pregnant women were not screened for *C*. *trachomatis* or *N. gonorrhoeae*. Laga, et al [15] in a study from Kenya, compared silver nitrate and tetracycline ointment for prophylaxis of ophthalmia in 2732 newborns. Pregnant women were tested for *C. trachomatis* and *N. gonorrhoeae* intrapartum; the results were not available before delivery. Ninety nine percent of infants who received silver nitrate and were exposed to maternal *C. trachomatis* infection were seen in follow-up clinics and 86% of infants who received tetracycline and were exposed to maternal *C. trachomatis* were seen in follow-up clinics [15].

It should be mentioned that Lund et al studied the incidence of gonococcal ophthalmia neonatorum (GON) in 23, 883 infants in Cape Town [24]; two prophylaxis agents (silver nitrate and erythromycin ophthalmic ointment) were introduced in routine eye care of the newborn [24]. Cases of GON decreased from 28 to 5 over the trial period [24]. However, this study was not used because they did not study Chlamydia ophthalmia [24].

Rours, et al from South Africa [17], followed 77 infants prospectively born to *C. trachomatis* positive mothers. Mothers were screened prenatally for *C. trachomatis* [17], however, there was no comparator, hence this study was not included in the final analysis. All infants received tetracycline ointment for neonatal prophylaxis; *C. trachomatis* conjunctivitis developed in 39% of infants born to *C. trachomatis* positive mothers [17].

### Included studies

The results of the three studies which met the four inclusion criteria are shown in Table 3.

**Table 3:** Included studies: Comparison of Efficacy of Neonatal Eye Prophylaxis for Prevention of *C. trachomatis* conjunctivitis

Study	Ref. No.	Prophylaxis	Odds ratio, 95% CI
Hammerschlag (1980)	**12**	Erythromycin v. Silver nitrate	0.06 [0.00, 0.33], *p* = 0.002*
Bell (1987)	**24**	Erythromycin v. Silver nitrate	0.60 [0.14, 2.04], *p* = 0.436
Hammerschlag (1989)	**3**	Erythromycin v. Silver nitrate	0.56 [0.23, 1.38], *p* = 0.206
		Tetracycline v. Silver nitrate	0.44, [0.14, 1.25], *p* = 0.104

* Statistically significant

Hammerschlag, et al [12], in a study conducted in Seattle in 1980 (*N* = 60 infants) demonstrated that neonatal ocular prophylaxis with erythromycin ophthalmic ointment decreased incidence of *C. trachomatis* ophthalmia neonatorum compared with silver nitrate (0 v. 33%, OR = 0.06, CI = [0.00, 0.33], *p* = 0.002). However, in a subsequent study conducted in Brooklyn in 1989 (*N* = 230 infants), Hammerschlag, et al [3] demonstrated that neonatal ocular prophylaxis with erythromycin or tetracycline ophthalmic ointments did not decrease incidence of *C. trachomatis* ophthalmia neonatorum compared with silver nitrate (erythromycin 14%: OR = 0.56, CI = [0.23, 1.38], *p* = 0.206, tetracycline 11%: OR = 0.44, CI = [0.14, 1.25], *p* = 0.104, v. 20% for silver nitrate). Bell, *et al* [23], from Seattle (*N* = 120 infants) demonstrated that neonatal ocular prophylaxis with erythromycin did not decrease the incidence of *C. trachomatis* conjunctiviti*s* compared to silver nitrate (15% v. 23%, OR = 0.60, CI = [0.14, 2.04], *p* = 0.436). Prophylaxis preparations were not randomized, infants received either preparation based on the preferences of the provider and/or parents.

## Discussion

The results of this study suggest that neonatal ocular prophylaxis with erythromycin or tetracycline ophthalmic ointments does not reduce incidence of neonatal chlamydial conjunctivitis or respiratory infection in infants born to mothers with *C. trachomatis* infection compared to silver nitrate. Prenatal screening and treatment of pregnant women is the most effective strategy for prevention of perinatal chlamydial infection, much as prenatal screening and treatment of gonococcal infection has been effective in preventing gonococcal ophthalmia. However, most countries worldwide do not routinely screen pregnant women for *C. trachomatis* [25].

Silver nitrate was first used for prophylaxis of gonococcal ophthalmia neonatorum in 1881 [1]. However, silver nitrate has not been available in the US for over two decades [4, 26, 27], and side effects include chemical conjunctivitis. The U.S. Preventive Task Force currently recommends neonatal ocular prophylaxis with erythromycin ophthalmic ointment for prevention of gonococcal, not chlamydial, ophthalmia [15]. Several countries in Europe (e.g. United Kingdom, Norway, Sweden, and Denmark) have discontinued universal ocular prophylaxis and others offer parental choice [27]. In 2015, the Canadian Pediatric Society recommended discontinuation of routine neonatal ocular prophylaxis in Canada with an emphasis on enhanced prenatal screening [26]. This policy was implemented in 2016 [8]. Erythromycin ophthalmic ointment has not been available in Canada for several years.

Only three studies met the inclusion criteria as described in the results. The initial 1980 study by Hammerschlag et al [12] yielded a significant result suggesting that ocular prophylaxis with erythromycin was effective in preventing neonatal chlamydial conjunctivitis compared to silver nitrate. However, the sample size was small (*N* = 60 infants). A subsequent larger study (*N* = 230 infants) conducted in Brooklyn, failed to confirm the initial results [3]. Even though the first study of Hammerschlag et al [12] was small, it yielded a highly significant result that induces replication; later larger studies of Hammerschlag et al [3] and Bell et al [23] failed to reproduce the early dramatic result. Sample size is often cited as the reason for why this occurs, and thus, one reason why systematic review is so important. Although Rours et al [17], was excluded from the final analysis because it was not comparative, the proportion of infants born to chlamydia positive mothers who received ocular prophylaxis with tetracycline ointment (39%) was similar to that reported in the comparative studies for erythromycin and tetracycline.

Erythromycin ophthalmic ointment is currently the only available FDA approved preparation in the U.S. for ophthalmia neonatorum. There is only one manufacturer and there have been interruptions in the supply. In 2009, the CDC informed physicians of a shortage of 0.5% erythromycin ointment due to change in manufacturers, and a set of interim guidelines were provided to clinicians of alternative agents (i.e. azithromycin or gentamicin ophthalmic preparations) [28]. However, no data on alternatives available for this indication and use of gentamicin ophthalmic ointment was associated with severe ocular reactions in the infants [28, 29].

We are aware of potential limitations of this study. This may include limitations at the study and outcome level (e.g. risk of bias) and/or at the review level (e.g. reporting bias and incomplete retrieval of identified research).

## Conclusion

This review highlights that the evidence of antibiotic prophylaxis for prevention of neonatal chlamydial conjunctivitis is sparse. However, screening and treatment of pregnant women is more effective than neonatal prophylaxis [30, 31].

## Data Availability

Data included in manuscript. The datasets used and/or analyzed during the current study available from the corresponding author on reasonable request.

## Abbreviations

NCC: Neonatal chlamydial conjunctivitis
GON: Gonococcal ophthalmia neonatorum
